# Immune‐Mediated Hepatotoxicity Associated With Ribociclib: A Multipatient Case Series and Management Strategy

**DOI:** 10.1155/crom/2417755

**Published:** 2026-08-03

**Authors:** Jemma Buchalter, Helen O′Donovan, Emmet Jordan, Zita Galvin, Janice M. Walshe

**Affiliations:** ^1^ Oncology Department, St. Vincent′s University Hospital, Dublin, Ireland; ^2^ Conway Institute, University College Dublin, Dublin, Ireland, ucd.ie; ^3^ Gastroenterology and Hepatology Department, St. Vincent′s University Hospital, Dublin, Ireland; ^4^ Oncology Department, University Hospital Waterford, Waterford, Ireland, hse.ie

## Abstract

Cyclin‐dependent kinase 4/6 inhibitors (CDK4/6i), when combined with endocrine therapy, have significantly transformed the management of hormone receptor‐positive, HER2‐negative metastatic breast cancer. Ribociclib, abemaciclib and palbociclib improve progression‐free survival and delay chemotherapy initiation, with ribociclib additionally demonstrating an overall survival advantage. The NATALEE trial has further expanded the role of CDK4/6 inhibition in early breast cancer, showing significant improvement in 4‐year invasive disease‐free survival with adjuvant ribociclib in HR+/HER2‐negative disease. Despite their shared mechanisms of targeting the CDK4/6–p16–retinoblastoma pathway, these agents differ in pharmacokinetics, kinase selectivity and toxicity. Hepatoxicity is a recognised adverse event of ribociclib with all grade toxicity occurring in around 20% of patients and Grade 3–4 events in 11%–15%. This drug‐induced liver injury (DILI) typically resolves with drug withdrawal.Immune‐mediated or autoimmune‐like presentations of ribociclib‐associated hepatotoxicity have been reported, although detailed management strategies remain poorly described. We report a series of five patients who developed ribociclib‐induced liver injury that failed to improve with cessation of therapy, and instead demonstrated features suggestive of an immune‐mediated mechanism. Hepatotoxicity occurred predominantly within the first few treatment cycles, highlighting the importance of early monitoring. All patients experienced significant biochemical improvement with corticosteroids; however, three developed recurrent transaminase elevation during tapering. Two required tacrolimus as a steroid‐sparing agent, allowing eventual withdrawal of immunosuppression without relapse. Rechallenge with ribociclib in one patient led to recurrent hepatotoxicity, again responsive to steroids. Three patients were subsequently treated with palbociclib without recurrence of hepatotoxicity, maintaining disease control for more than 2 years. Liver biopsy in four patients was consistent with DILI in three and revealed mixed DILI with underlying primary biliary cholangitis in one, illustrating the complexity of managing hepatotoxicity in individuals with comorbid liver disease. This series highlights a pattern suggestive of immune‐mediated ribociclib‐induced hepatotoxicity responsive to short course corticosteroids with or without additional immunosuppression. Palbociclib appears to be a safe alternative, enabling continuation of CDK4/6–targeted therapy without premature transition to chemotherapy.

## 1. Introduction

The development and introduction of cyclin‐dependent kinase 4/6 inhibitors (CDK4/6i) as the new standard of care, in combination with endocrine therapy (ET), has significantly changed the treatment landscape for hormone receptor‐positive (HR+), HER2‐negative (HER2−) metastatic breast cancer (MBC) [1, 2]. Three CDK4/6i have been approved by both the FDA and EMA: ribociclib, abemaciclib and palbociclib. All three drugs have demonstrated improvement in progression‐free survival (PFS), response rate (RR) and consistently delay chemotherapy initiation in HR+/HER2– MBC in both first‐ [3–7] and second‐line treatment [8–10] compared with ET alone. However, only ribociclib has demonstrated a statistically significant overall survival benefit in the first line setting in Phase III clinical trials for HR+/HER2− MBC [3–10]. The role of CDK4/6 inhibition has been expanded into the adjuvant setting for early breast cancer, with abemaciclib and ribociclib both approved [11, 12]. The recently published NATALEE trial demonstrated a significant improvement in 4‐year invasive disease‐free survival with adjuvant ribociclib [12].

All three CDK4/6 inhibitors are oral, small molecules that selectively inhibit both cyclin D‐cyclin dependent kinase (CDK) 4 and 6. They exert their action via the CDK4/6–p16–retinoblastoma pathway and are a key regulator of the G1 to S phase cell cycle checkpoint ensuring appropriate cell growth and proliferation. In some malignancies, such as breast cancer, oncogenic mutations can result in upregulation of this pathway, specifically at CDK4 and CDK6, making these attractive therapeutic targets [13]. CDK4/6 inhibitors, although in the same class of drug, exert differences in terms of pharmacokinetics, target selectivity and toxicities that are likely attributable to the differences in affinity towards CDK4 versus CDK6 by each of the drugs [13].

The SPC for ribociclib advises that liver function tests (LFTs) should be performed before initiating treatment, every 2 weeks for the first two cycles, at the beginning of each of the subsequent four cycles and then as clinically indicated. A Grade 2, or greater, liver toxicity requires the medication to be held until LFTs return to baseline with a subsequent dose reduction if a Grade 3 or higher elevation is observed (five times the upper limit of normal [ULN] of alanine aminotransferase [ALT] or aspartate transferase [AST]) [14]. According to the comprehensive database LiverTox, in preregistration clinical trials, all grade ALT elevations occurred in 46% of patients treated with ribociclib versus 36% of control subjects and Grade 3 elevations, in 10% versus 1%, respectively [15]. However, the large randomised control trials, such as MONALEESA‐2, demonstrated significantly lower rates of hepatotoxicity. All grade ALT elevation occurred in 20.4% of patients receiving ribociclib compared with 6.4% in the placebo arm, whereas Grades 3–4 elevation occurred in 11.4% compared with 1.2%, respectively. The incidence of Grades 3–4 ALT elevation was slightly lower in the MONALEESA‐3 and 7 trials, at 13.7% and 11%, respectively [3, 4, 8, 16]. Although recovery of LFTs was slow in some cases, taking 3 to 5 months, complete resolution was observed in all cases. Immunoallergic and autoimmune features were not reported. In the adjuvant setting, rates of LFTs derangement were similar, with 19.7% experiencing all grade ALT elevation and 7.7% experiencing Grades 3–4 elevation, despite use of a lower dose of ribociclib [12].

Although hepatotoxicity associated with ribociclib is well‐recognised, it is typically self‐limiting and resolves with drug interruption alone [3, 4, 9, 16], a subset of cases may demonstrate features suggestive of an immune‐mediated drug‐induced liver injury (DILI), demonstrating persistence despite drug withdrawal and responsiveness to corticosteroids, a phenomenon not routinely described with CDK4/6 inhibitors.

We describe a series of five patients with ribociclib‐associated liver injury characterised by persistent biochemical deterioration despite drug withdrawal, sresponsiveness to steroids (with rebound on tapering in some cases) and, in selected cases, a requirement for steroid‐sparing immunosuppression. These features suggest an immune‐mediated mechanism, with important implications for ongoing CDK4/6 inhibitor therapy. All five cases are summarised in Table 1.

**Table 1:** Summary of hepatic injury and management: ALT values represent peak alanine aminotransferase levels observed during acute injury.

Case	Cycle of onset	Peak ALT (IU/L)	Peak bilirubin (*μ*mol/L)	Liver biopsy	Improvement after ribociclib withdrawal alone	Steroid response/treatment	LFT rebound on taper	Additional immunosuppressant	Time to LFT normalisation	Rechallenge outcome	Subsequent CDK4/6i
1	Cycle 2	1628	104	Yes—DILI with CD3/CD8 lymphocytic infiltrate	No	Yes (rapid) prednisolone 60 mg	No	No	~2 months	Not attempted	Palbociclib (1 yr)
2	Cycle 2	2058	135	Yes—lobular hepatitis/DILI	No	Yes prednisolone 60 mg	Yes	Tacrolimus	~4 months	Not attempted	Palbociclib (> 2 yrs)
3	Cycle 4	519	Normal	No	No	Yes prednisolone 40 mg	Yes	No	2–3 months	Recurrence of hepatotoxicity	Palbociclib (> 1 yr)
4	Cycle 3	100	154	Yes—PBC with superimposed DILI features	No	Yes prednisolone 30 mg	No	No	Partial improvement only	Not attempted	No—fulvestrant only
5	Cycle 3	2553	44	Yes—severe portal lymphohistiocytic inflammation favouring DILI	No	Yes IV methylprednisolone then oral prednisolone	Yes	Tacrolimus	~4 months	Not attempted	No—single agent tamoxifen only

### 1.1. Case 1

In 2014, a 68‐year‐old Caucasian female presented with a left sided breast mass and was diagnosed with a HR+, HER2‐negative breast cancer. Left sided mastectomy and axillary lymph node clearance confirmed a pT2N3, Grade 3, invasive ductal carcinoma with 31/35 positive lymph nodes. Treatment with adjuvant chemotherapy including dose dense Adriamycin, cyclophosphamide and paclitaxel was administered, followed by radiotherapy and ET using anastrozole. In 2021, while compliant on ET, the patient relapsed with biopsy proven metastatic HR+, HER2− breast cancer with bilateral lung deposits and mediastinal lymph nodes.

The patient was started on fulvestrant and ribociclib (600 mg, 21 days on/7 days off, of a 28‐day cycle). On initiation of ribociclib, LFTs were normal. Cycle 2 Day 15 blood work demonstrated Grade 3 hepatocellular injury (alanine transaminase [ALT] 598 IU/L and AST 465 IU/L) with normal bilirubin and INR. Despite cessation of ribociclib, transaminases continued to rise, peaking at ALT 1628 IU/L, AST 2251 IU/L and bilirubin 104 IU/L. The patient displayed no clinical signs of liver dysfunction. Investigations including liver ultrasound, CT liver imaging, viral hepatitis screen, autoimmune serology and medication review did not identify an alternative cause. A nontargeted liver biopsy was performed demonstrating significant inflammatory infiltrates with CD3/CD8 positive lymphocytes and necrosis, consistent with DILI following multidisciplinary review. Despite ribociclib being discontinued for 19 days, LFTs continued to rise. Prednisolone was started at 60 mg daily, with significant improvement in transaminases noted within 48 hours. Bilirubin continued to rise until Day 5 of steroids, after which the bilirubin improved. Prednisolone was successfully tapered and weaned over a period of 2 months with no further deterioration in LFTs. Palbociclib was commenced when LFTs returned to baseline for management of her MBC, and no hepatotoxicity was observed. Palbociclib was safely continued for 1 year until the patient developed progressive disease.

### 1.2. Case 2

A 60‐year‐old female was initially diagnosed with right‐sided HR+, HER2−, lymph node‐positive breast cancer in 2011. She had a wide local excision (WLE) and sentinel lymph node biopsy (SLNB), which identified a pT1cN1, Grade 3, invasive ductal carcinoma. She was then treated with six cycles of docetaxel and cyclophosphamide followed by radiotherapy and 5 years of tamoxifen. Ten years after initial treatment the patient was diagnosed with metastatic HR+, HER2− breast cancer to lung. She was started on ribociclib (600 mg, 21 days on and 7 days off, of a 28‐day cycle) with letrozole.

On Cycle 2 Day 1, LFTs were noted to be elevated; AST 104 IU/L (Grade 1 toxicity) and ALT 222 IU/L (Grade 2). Ribociclib was held but letrozole continued. Despite cessation of ribociclib, liver biochemistry worsened with peak AST 1255, ALT 2058, GGT 320, ALP 312 and bilirubin 40 IU/L. The patient was admitted for further investigations, including liver ultrasound, viral hepatitis serology, autoimmune liver screen and medication review, which did not identify an alternative aetiology. Liver biopsy demonstrated lobular hepatitis with lymphocytic infiltrates, no evidence of chronic liver disease and negative stains for amyloid (Perls stain), copper associated protein (Shikata stain) and alpha‐1‐antitrypsin (PAS stain), favouring DILI. This case was discussed at MDT and felt most likely to be consistent with DILI. Given that there was no improvement in LFTs, despite holding ribociclib, prednisolone 60 mg daily was commenced. Transaminases improved immediately. Bilirubin continued to climb after initiation of steroids, peaked at 135 IU/L, but then rapidly returned to normal. A slow steroid taper was commenced, but when prednisolone was reduced to 20 mg, a rebound Grade 3 increase in transaminases was observed, ALT 530 and AST 258 IU/L. On reintroduction of 30 mg prednisolone, transaminases improved. Tacrolimus was subsequently commenced as a steroid sparing agent, allowing successful tapering of steroids, with normalisation of LFTs within 4 months of initial DILI onset. The target tacrolimus level was 3–5. The patient experienced some mild headaches and tremor while on tacrolimus. The patient′s breast cancer treatment was changed to palbociclib with the same ET and tacrolimus continued for the first 3 months of treatment. The patient had a mild increase in LFTs during this period, ALT 60 from 23 IU/L and AST 44 from 24 IU/L, so her Tacrolimus dose was increased slightly and LFTs settled. After 3 months of palbociclib, tacrolimus was successfully weaned over a period of 6 weeks. Two years later, the patient remains on palbociclib with stable metastatic disease and no further events of hepatotoxicity.

### 1.3. Case 3

A 44‐year‐old Caucasian female presented in 2018 with a palpable breast mass and multiple axillary lymph nodes. She was diagnosed with a left sided multifocal, HR+, HER2−, lymph node‐positive breast cancer. She had a left sided WLE and SLNB (pT2(m)N1) followed by adjuvant chemotherapy, six cycles of Taxotere and cyclophosphamide, radiotherapy and tamoxifen. Compliance with tamoxifen was poor due to intolerance and was eventually discontinued 18 months after initiation. In 2024, CT imaging detected new suspicious pulmonary lesions, and a VATS biopsy confirmed metastatic HR+ HER2− breast cancer. Treatment was initiated with ribociclib (600 mg, 21 days on/7 days off, 28‐day cycle), anastrozole and zoladex.

LFTs initially demonstrated Grade 1 transaminitis on Cycle 3 Day 1 (ALT 59 and AST 48 IU/L). By Cycle 4 Day 1, ALT had increased to 162 IU/L (Grade 2) and AST 88 IU/L (Grade 1), prompting ribociclib interruption. One week later, despite drug cessation, liver injury progressed with peak ALT 519 IU/L and AST 357 IU/L. The patient had no clinical signs of hepatic dysfunction. Viral hepatitis screen,, liver ultrasound, medication review and coagulation studies were unremarkable, aside from a positive anti‐CMV IgG, consistent with past exposure rather than active infection. The patient had no significant past medication history. Prednisolone was commenced at 40 mg, with a substantial reduction in transaminases, allowing steroid weaning ?added value="to be"?> successfully commenced.

LFTs were consistent with Grade 1 hepatitis three weeks poststeroid commencement (ALT 111 IU/L and AST 52 IU/L). Ribociclib was recommenced at a level‐one dose reduction (400 mg), alongside a tapering dose of prednisolone.

On Cycle 1 Day 14 of dose‐reduced ribociclib (Cycle 5 in total), lab investigations were performed. At this time, steroids had been discontinued for 4 days. Repeat LFTs demonstrated a rebound rise in transaminases (ALT 244 IU/L and AST 103 IU/L), ribociclib was stopped and prednisolone recommenced at 25 mg. Initially, LFTs improved but a second rebound rise in LFTs was demonstrated on attempted steroid tapering. A higher dose of steroid was recommenced (30 mg, daily) and successfully weaned at a slower rate, resulting in LFTs returning to baseline. Once liver biochemistry had normalised the patient was transitioned to palbociclib with the same endocrine backbone, without evidence of recurrent hepatotoxicity.

### 1.4. Case 4

A 48‐year‐old female underwent bilateral breast cancer surgery in December 2022 for leftsided HR+, HER2−, lymph node‐positive pleomorphic lobular breast cancer (pT3N1) and a right‐sided HR+, HER2− lobular (pT1cN0) breast cancer. She had a background history of primary biliary cholangitis (PBC) and had been on treatment with ursofalk, rifampicin, fexofenadine and naltrexone. Imaging performed postsurgery demonstrated oligometastatic disease to T12 in January 2023. She underwent radiotherapy to T12 and then started on fulvestrant and ribociclib 600 mg. Following Cycle 3 of ribociclib, the patient developed an acute cholestatic liver injury with a bilirubin 154, ALP 1719, GGT 1562, ALT 31 and AST 96 IU/L. Liver imaging excluded metastatic progression or biliary obstruction. Liver biopsy demonstrated previously known PBC with superimposed eosinophilic and inflammatory changes considered consistent with DILI following multidisciplinary review. She was started on 30 mg of prednisolone with a plan to slowly taper. Within 1 month, LFTs had improved to a bilirubin 112, ALP 975, GGT 841, ALT 35 and AST 69 IU/L. By 2 months, prednisolone had been weaned to 10 mg. Bloods remained stable but did not normalise; bilirubin was 98, ALP 200 and ALT 100 IU/L. At time of writing, ribociclib was not reintroduced, and the patient continues on single‐agent fulvestrant with no evidence of disease progression.

### 1.5. Case 5

In March 2023, a 51‐year‐old female presented with abdominal discomfort and nausea and was subsequently diagnosed with Stage IV breast cancer affecting the breast and liver. Breast histology demonstrated an invasive lobular carcinoma (ILC), Grade 2, HR+, HER2‐. She was started on Ovarian function suppression (OFS)/letrozole/ribociclib (600 mg, 21 days on/7 days off). On Cycle 2 Day 1 of treatment, ribociclib was dose reduced to 400 mg due to prolonged QTc. Cycle 3 Day 1 blood tests demonstrated a severe hepatocellular injury with ALT 1572 IU/L, subsequently peaking at 2553 IU/L (Grade 4 hepatitis), with a bilirubin of 20.5 IU/L. Ribociclib, letrozole and OFS were held with no improvement in LFTs observed. Prednisolone 40 mg was commenced and her ALT reduced to 526 IU/L after 4 weeks.

Two attempts were made to taper the steroids, but LFTs increased on both occasions. On the failed second taper, she was admitted to hospital for further investigation, including liver biopsy; at this time ALT peaked at 1315 IU/L (Grade 4) and bilirubin was 44.4 IU/L. During this admission, high dose IV methylprednisolone was commenced, resulting in a significant improvement in LFTs.

Extensive viral and autoimmune investigations were performed. Initial hepatitis E serology (Hep E IgM) was equivocal, with subsequent discordant serology (positive Hep E IgM and IgG) but persistently negative HEV viral load. Her steroids were quickly tapered given this result but unfortunately transaminases increased again. Following discussion with the National Virus Reference Laboratory, these findings were considered noncontributory and most consistent with false‐positive serology. IV steroids were increased following the transaminase rise,then switched to a slow tapering regimen of oral prednisolone. Within 4 weeks ALT had reduced to 289 IU/L and bilirubin had returned to baseline. Liver biopsy demonstrated severe portal‐based inflammation, which was predominantly lymphohistiocytic; both plasma cells and eosinophils were also seen, favouring DILI over viral hepatitis. Steroids were increased following MDT discussion, which concluded that histology favoured DILI and that the repeated infectious screen nonconcerning. Prednisolone dose was increased to 30 mg from 10 mg, and tacrolimus 1 mg BD was added, aiming for a level of 3–5 ng/mL. Steroids were slowly tapered, and LFTs returned to baseline by December 2023 (ALT 40 IU/L and bilirubin 9.8 IU/L). Tacrolimus was successfuly discontinued after 3 months of therapy and liver biochemistry subsequently normalised, and the patient was transitioned to single agent tamoxifen.

## 2. Discussion

DILI is traditionally classified as intrinsic or idiosyncratic. Intrinsic or direct DILI is predictable and occurs in a significant proportion of individuals exposed to the drug. It is typically dose‐related with onset within a short time span. Paracetamol toxicity is a typical example of this. Idiosyncratic DILI is usually not dose‐related and occurs in a small proportion of individuals. It is variable in dosing, time of onset and duration. The adaptive immune system plays a role in idiosyncratic DILI. However, these patients often do not exhibit systemic allergic features such as rash or eosinophilia. Immunotherapeutic agents, including biologics, and in particular immune checkpoint inhibitors for advanced cancer, are associated with immune‐mediated adverse reactions, including liver injury [17].

In general, CDK4/6 inhibitors are a very well‐tolerated class of drugs, but all have notable toxicities. Palbociclib is associated with high levels of myelosuppression, abemaciclib with diarrhoea, and ribociclib with liver toxicity and QTc prolongation [3, 5, 6]. All three MONALEESA trials( MONALEESA ‐2, ‐3 and ‐7) describe an increased incidence of Grade 3 and 4 hepatotoxicity with ribociclib compared with placebo, with the incidence rate of Grade 3 hepatobiliary toxicity quoted at 15.6%, 13.7%, and 11.0%, respectively [3, 4, 8]. LiverTox suggests that the causes of serum enzyme elevations and liver injury from ribociclib therapy are not known. Ribociclib is extensively metabolised in the liver largely through the CYP 3A4 pathway, and liver injury might be caused by production of a toxic or immunogenic intermediate. On the other hand, inhibition of cyclin‐dependent kinases 4 and 6 may also affect hepatocytes and have direct toxicity. The hepatotoxicity usually seen with ribociclib resolves on withdrawal of the drug [15].

All cases underwent structured evaluation for alternative causes of liver injury. Investigations included viral hepatitis screening (including Hepatitis A, B, C and E, with CMV and EBV testing where clinically indicated), autoimmune liver serology, liver imaging and medication review for alternative hepatotoxic agents. Liver biopsy was performed in four cases. In all patients, the association of ribociclib exposure, worsening LFTs despite drug withdrawal, exclusion of competing aetiologies and subsequent corticosteroid responsiveness supported a diagnosis of ribociclib‐associated DILI with immune‐mediated features. Although formal causality assessment tools such as RUCAM [18] may strengthen reproducibility, the retrospective nature of this series and complexity of oncology‐associated liver injury limited their application.

The cases outlined in this report suggest that some presentations of ribociclib‐associated DILI may involve immune‐mediated features. Each case failed to respond to withdrawal of ribociclib, which we would routinely expect, and no other cause for liver injury was identified on investigation. Initiation of steroids in each case resulted in a rapid improvement in all LFTs; however, bilirubin levels were slower to respond than the transaminases when elevated. In three cases, tapering of steroids resulted in a rebound increase in LFTs, which responded to reintroduction of a higher dose of steroids. In two of these cases, tacrolimus was introduced as a steroid‐sparing agent, and both steroids and tacrolimus were successfully weaned with no rebound phenomena witnessed. There ise no data on the use of tacrolimus or on the ideal target levels in this setting. In the two cases, tacrolimus was added as the LFTs had been difficult to control as steroids were tapered. In both cases, the tacrolimus levels were kept below 5 ng/mL, and the tacrolimus was stopped as quickly as possible (in both cases within 3 months). In the third case, once steroids had been weaned, ribociclib was reintroduced at a dose reduction and a recurrence of elevated LFTs was observed. This required repeated discontinuation of ribociclib and reintroduction of steroids, with a slower steroid wean, this was successful and LFTs returned to baseline.

This response to steroids +/− tacrolimus, with rebound observed on tapering, further suggests a possible immune‐mediated mechanism associated with this type of liver injury.

Although management was individualised, several consistent clinical features emerged across cases. Corticosteroids were introduced in patients with progressive biochemical liver derangement despite ribociclib withdrawal, given that transaminases continued to rise after drug cessation and alternative causes had been excluded. Initial prednisolone doses ranged from 30 to 60 mg daily depending on the severity of hepatitis and bilirubin elevation. In patients demonstrating rebound hepatitis during tapering, a slower corticosteroid taper was employed and tacrolimus was introduced selectively as a steroid‐sparing strategy. Based on this experience, we propose that persistent or worsening hepatitis despite withdrawal of ribociclib should prompt early hepatology review, consideration of liver biopsy and evaluation for DILI with immune‐mediated features.

In three of the cases, palbociclib was successfully commenced as the next line of treatment for a sustained period with no recurrence of hepatic dysfunction, the longest duration of treatment being 2 years and ongoing in this series. These observations suggest that palbociclib may represent a feasible alternative in selected patients, further delaying escalation to chemotherapy.

Four of the patients underwent liver biopsy. Following discussion at MDT, these were deemed consistent with DILI in the clinical setting in three cases. DILI was seen to contribute to the histological findings along with typical PBC features in one case. The case of the patient with PBC also highlights the difficulties of managing liver drug toxicities in patients with an underlying liver disease. In this case, an initial drug toxicity was observed on Cycle 2 (QTc prolongation) with a second developing on Cycle 3 (liver toxicity). These cases should be managed in a centre with specialist hepatology input.

Liver injury was usually witnessed within the first few cycles of treatment (two in the second cycle, two in the third and one in the fourth). The increased frequency of blood checks in the first two cycles allows for earlier detection of LFT derangement and earlier management. However, immune‐mediated liver injury can be experienced at any time during CDK4/6 inhibitor treatment, so careful monitoring of LFTs at the start of each treatment cycle is required and clinicians should remain alert to the possibility of immune‐mediated DILI throughout treatment. This case series also highlights the importance of multidisciplinary team involvement: oncology, hepatology, pathology, and radiology, in managing these complex cases.

Emerging reports have described autoimmune‐like or immune‐mediated ribociclib‐induced liver injury, including cases characterised by steroid responsiveness and histological findings resembling autoimmune hepatitis [19]. Our findings are consistent with these observations but additionally describe recurrent hepatitis during steroid tapering, successful use of tacrolimus as a steroid‐sparing agent in selected patients, and subsequent successful treatment with palbociclib in several cases.

## 3. Conclusion

This case series highlights that ribociclib‐associated hepatotoxicity may, in selected cases, demonstrate immune‐mediated features, including persistence despite drug withdrawal, corticosteroid responsiveness, and biochemical rebound during steroid tapering. Some patients required additional immunosuppression with tacrolimus to facilitate steroid withdrawal. These findings support consideration of immune‐mediated DILI in patients with progressive liver injury despite ribociclib cessation. In selected patients, palbociclib appeared to be a feasible alternative CDK4/6 inhibitor without recurrent severe hepatotoxicity. Larger studies are required to better define the incidence, optimal management, and long‐term outcomes of this presentation.

## Author Contributions

All authors have made substantial contributions to the conception of the report and to drafting or revising it for important intellectual content.

## Funding

No funding was received for this manuscript.

## Ethics Statement

All patient information has been anonymised. Both verbal and written consent was obtained.

## Conflicts of Interest

The authors declare no conflicts of interest.

## Data Availability

The data that support the findings of this study are available on request from the corresponding author. The data are not publicly available due to privacy or ethical restrictions.
